# Endodermal Sinus Tumor of the Neck: A Rare Presentation

**DOI:** 10.7759/cureus.45630

**Published:** 2023-09-20

**Authors:** Ankita Gyanchandani, Samarth Shukla, Sunita Vagha, Sourya Acharya, Keyur Saboo

**Affiliations:** 1 Department of Pathology, Jawaharlal Nehru Medical College, Datta Meghe Institute of Higher Education and Research (Deemed to be University), Wardha, IND; 2 Department of Medicine, Jawaharlal Nehru Medical College, Datta Meghe Institute of Higher Education and Research (Deemed to be University), Wardha, IND

**Keywords:** histopathology, chemotherapy, case report, fine needle aspiration cytology (fnac), malignant germ cell tumour

## Abstract

Germ cell tumors usually occur in the gonads. Extragonadal germ cell tumors (EGCTs) are uncommon, and the head and neck region is a rare primary location. In this region, endodermal sinus tumors are relatively uncommon; they are typically recorded alongside teratomas and infrequently by themselves. When an endodermal sinus tumor arises in the neck, it can present with unique clinical and radiographic features and can be challenging to diagnose and manage. We report the peculiar case of a 25-year-old young adult who was suffering from a painful left-sided neck swelling for a year. Fine needle aspiration cytology (FNAC) of neck mass was performed and the cytomorphological features were suggestive of germ cell tumor. After the diagnosis, the patient received chemotherapy and was posted for wide local excision of the tumor. On microscopic examination, the tumor mass showed histopathological features suggestive of an endodermal sinus tumor.

## Introduction

Germ cell tumors (GCTs) encompass a category of benign and malignant tumors, mostly afflicting the gonads of children and young males with histologic and behavioral heterogeneity. Extragonadal GCTs (EGCTs) are rare, and non-gonadal sites, particularly the mediastinum and retroperitoneum, account for 5-10% of all GCTs [1]. Endodermal sinus tumors are gonadal site-derived GCTs. Sacrococcygeal, mediastinal, intracranial, and retroperitoneal regions are the most prevalent locations for extragonadal endodermal sinus tumors. Endodermal sinus tumors are very unusual in the extracranial head and neck, and only a few cases are reported [2]. The head and neck are rarely affected comprising about 1% of all cases. Treatment usually involves surgery to remove the tumor, followed by chemotherapy and radiation therapy. The prognosis depends on various factors such as the extent of the tumor and whether it has spread to other parts of the body [3]. In this study, we report an uncommon cause of endodermal sinus tumor of the neck in a young adult presenting certain characteristic features of endodermal sinus tumor in the head and neck.

## Case presentation

A 25-year-old young male presented with painful neck swelling for one year. The swelling was initially small in size, approximately lemon-sized. The swelling was gradual in onset and associated with tenderness. There was no loss of weight and no appetite loss. On clinical examination, a 7 x 4 cm swelling was noted in the neck region at level III and IV lymph nodes. The swelling was firm to tender hard. Systemic examination was within normal limits. Serum alpha-fetoprotein was 1032 ng/ml and serum beta-human chorionic gonadotropin (hCG) was 8975 mIU/m

**Figure 1:**
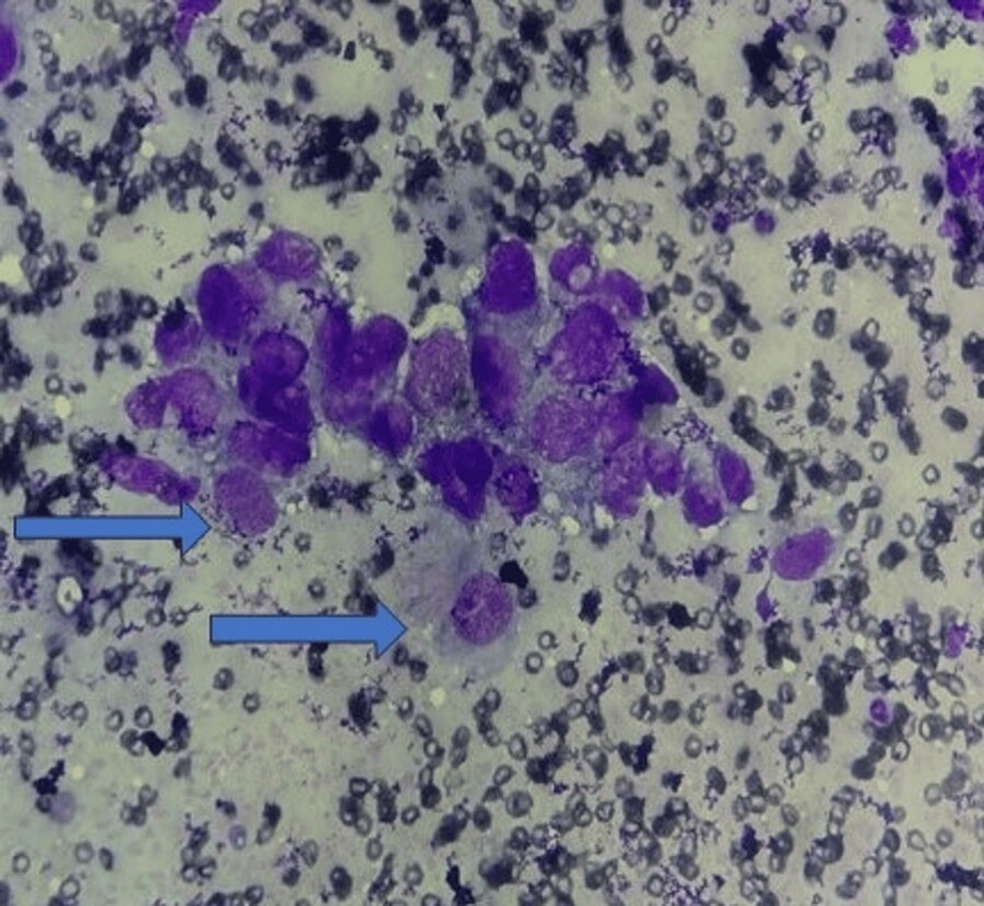
FNAC revealed cells with ovoid nuclei with irregular nuclear border, clumped chromatin. Cytoplasm of the cells was abundant with few cells showing intracytoplasmic inclusions (Giemsa stain 40x) (blue arrow) FNAC: fine needle aspiration cytology

Fine needle aspiration cytology (FNAC) from the lesion revealed cells with ovoid nuclei with irregular nuclear borders and clumped chromatin. Cytoplasm of the cells was abundant with few cells showing intracytoplasmic inclusions. Based on cellular features, a diagnosis of malignant germ cell tumor was made; however, Schiller Duval bodies were not identified (Figure 1).

**Figure 2:**
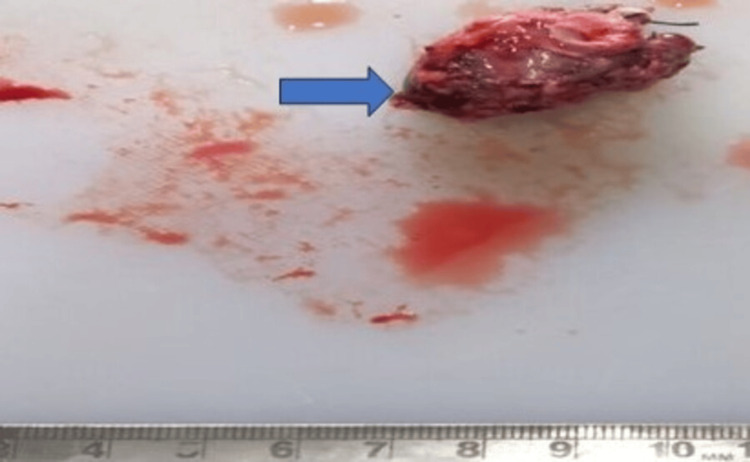
Gross specimen of tumor specimen of the neck showing single, irregular, brownish-red tissue piece measuring 3.5 x 3 cm (blue arrow). On the cut section, hemorrhagic areas were identified.

A positron emission tomography (PET) scan was done to rule out metastasis. The patient received four cycles of chemotherapy with etoposide, bleomycin, and cisplatin. After chemotherapy, the patient’s beta-hCG was 15.57 ng/ml and serum alpha-fetoprotein level was 1.75 ng/ml. Six months after chemotherapy and after obtaining all required fitnesses, the patient was posted for wide local excision of the tumor including left-sided modified radical neck dissection. A grossly single, irregular brownish-reddish tissue piece measuring 3.5 x 3 cm was received (Figure 2).

**Figure 3:**
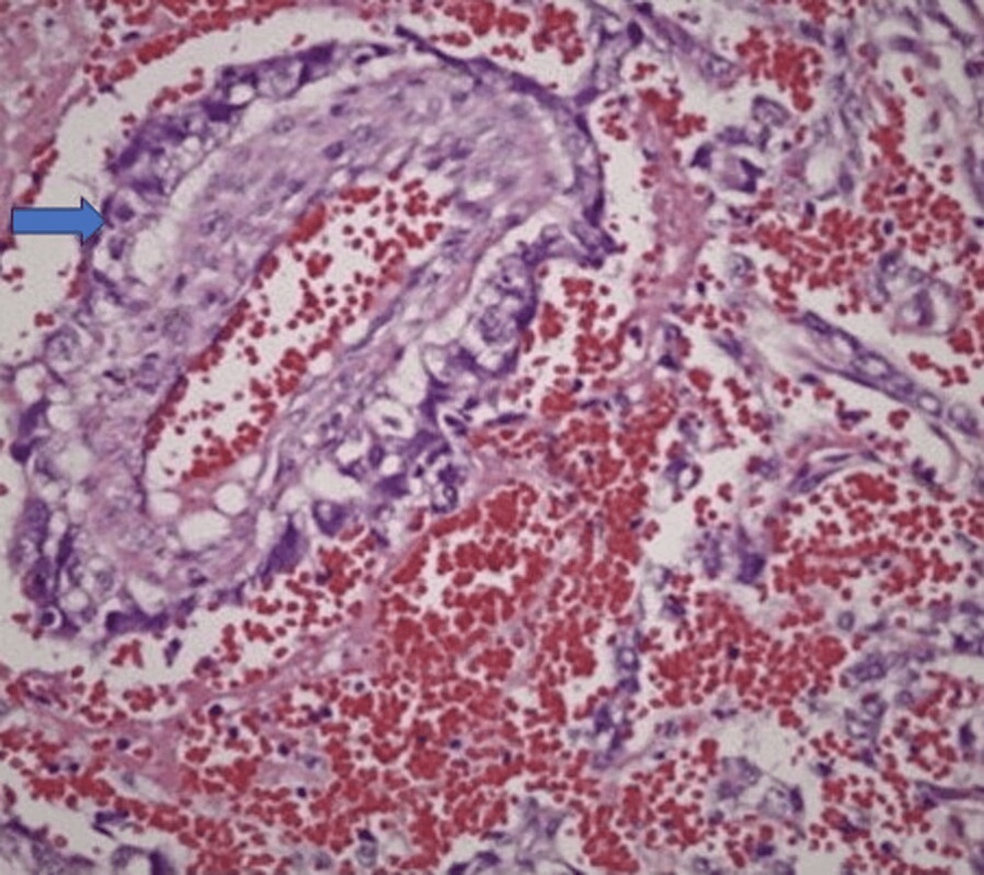
H&E staining (40 x) of specimen showing characteristic Schiller Duval bodies (blue arrow).

On microscopic examination, the tumor mass showed an endodermal sinus growth pattern and microcystic spaces interlined with cuboidal to polygonal cells with a moderate amount of eosinophilic cytoplasm, vesicular nuclei, and conspicuous nucleoli. A distinctive Schiller Duval body was seen without any coexisting features of teratoma showing histopathological features suggestive of an endodermal sinus tumor (Figure 3), and it was positive for vascular and perineural invasion. Also, the proximal margin and lateral margin showed infiltration by tumor cells on histopathology.

**Figure 4:**
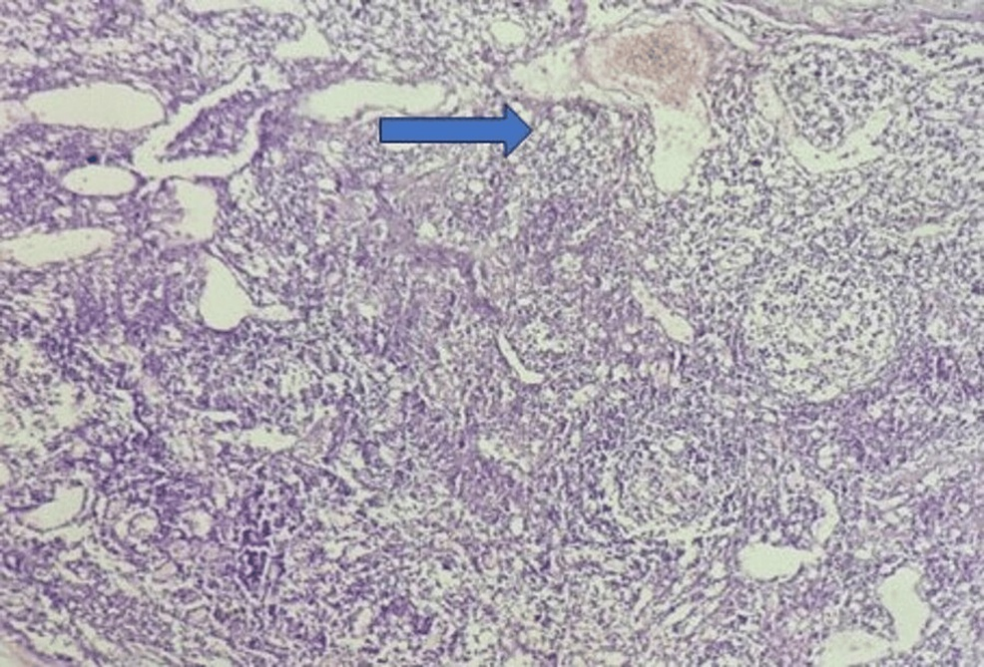
H&E (10x) staining showing lymph node positive for infiltration by malignant cells (blue arrow).

Twenty lymph nodes were identified, out of which 17 showed infiltration by tumor cells on histopathology (Figure 4).

## Discussion

The most reasonable explanation for the endodermal sinus tumor, notwithstanding the different theories that have been proposed so far, still revolves around the dissociation of germ cells from the gonadal crest during embryogenesis and the subsequent malignant transformation of these cells.

Although rare cases have also been known to occur in extragonadal sites like the vulva, vagina, pineal region, wide ligament, prostate, cervix, mediastinum, sacral-coccygeal region, and retroperitoneal area, endodermal sinus tumors are mostly reported in the testes and ovaries [4,5]. According to the MAKEI (Maligne Keimzelltumoren) group, the largest group that has investigated GCTs of the head and neck, endodermal sinus tumors in the head and neck make up 1% of all malignant GCTs [6].

Extragonadal endodermal sinus tumor is difficult to diagnose, particularly in tiny specimens. Low histological suspicion is frequently a result of low clinical suspicion, particularly in non-midline tumors. This situation is made worse by the wide variety of morphological manifestations of yolk sac tumors (YSTs). Ten microscopic patterns, consisting of microcystic, macrocytic, solid, glandular, papillary, endodermal sinus, myxomatous, poly vesicular vitelline, hepatoid, and enteric patterns, are officially recognized by the World Health Organization. Additionally, the majority of neoplasms display a variety of patterns [1].

Endodermal sinus tumors are a secondary cause of elevated serum alpha-fetoprotein levels. However, elevated values are not sufficient for diagnosis as is the case with immunostaining of tumor cells by alpha-fetoprotein antibody, as this abnormality may be seen in other tumors such as hepatocellular and pancreatic carcinomas. O-fetoprotein concentrations can be tested before and after treatment since they are a reliable indicator of treatment response, recurrence, and metastatic illness [7].

Head and neck endodermal sinus tumors are relatively uncommon. In a case comparable to this one, Jeyasakthy et al. described a study in which there was the malignant transformation of mature teratoma into a YST of the neck in an infant [4]. Following histological analysis of the neck mass and a significant rise in serum alpha-fetoprotein, the diagnosis was confirmed. Sadly, the child passed away before receiving treatment. Very few studies of endodermal sinus tumors of the head and neck have been documented so far.

At diagnosis, EGCTs in the head and neck are generally not resectable. Particularly aggressive endodermal sinus tumors have a significant likelihood of local recurrence and/or early spread. For malignant EGCTs in the head and neck, an interdisciplinary approach with a curable goal through excision by surgery and adjuvant chemotherapy is typically advised. Bleomycin, etoposide, and cisplatin, collectively known as the BEP regimen, are a trio of multi-agent chemotherapy drugs that have considerably improved the prognosis of EGCT patients [1].

## Conclusions

Endodermal sinus tumor of the head and neck region is an extremely rare entity, and its clinical features, histological characteristics, and treatment outcomes have not been extensively studied. Extragonadal endodermal sinus tumors particularly in the head and neck, are uncommon with rarer clinical suspicion making the histopathological diagnosis a difficult task. However, a primary and accurate diagnosis is required for developing the optimal therapeutic plan.
